# Integrated Design of Antibodies for Systems Biology Using *Ab Designer*

**DOI:** 10.4172/jpb.1000307

**Published:** 2014-03-24

**Authors:** Trairak Pisitkun, Patrick Dummer, Poorichaya Somparn, Nattiya Hirankarn, Jeffrey B Kopp, Mark A Knepper

**Affiliations:** 1 Epithelial Systems Biology Laboratory, National Heart, Lung and Blood Institute, NIH, Bethesda, MD, USA; 2 Kidney Disease Section, National Institute of Diabetes and Digestive and Kidney Diseases, NIH, Bethesda, MD, USA; 3 Faculty of Medicine, Chulalongkorn University, Bangkok, Thailand

**Keywords:** Antibody design, Software tools, Systems biology

## Abstract

In the current era of large-scale biology, systems biology has evolved as a powerful approach to identify complex interactions within biological systems. In addition to high throughput identification and quantification techniques, methods based on high-quality mono-specific antibodies remain an essential element of the approach. To assist the large-scale design and production of peptide-directed antibodies for systems biology studies, we developed a fully integrated online application, *AbDesigner* (http://helixweb.nih.gov/AbDesigner/), to help researchers select optimal peptide immunogens for antibody generation against relatively disordered regions of target proteins. Here we describe *AbDesigner* in terms of its features, comparing it to other software tools, and use it to design three antibodies against kidney disease-related proteins in human, viz. nephrin, podocin, and apolipoprotein L1.

## Introduction

The function of individual cells in mammals depends on which of the >20,000 protein coding genes are expressed and on regulation of the properties of the expressed proteins within the cell. A major goal in systems biology applied at a cellular level is to identify what proteins are expressed in a given cell type, to measure certain attributes of the expressed proteins, and to model cellular functions based on this information [1,2]. Critical attributes of proteins that must be measured include: 1) the abundance of each protein, 2) the subcellular distribution of each protein, 3) the post-translational modifications (PTMs) present in each protein, and 4) the binding interaction that each protein participates in. Because of its ability to measure these attributes at a large-scale, i.e. across the proteome, protein mass spectrometry has taken a leading role in systems biology discovery and quantification [3,4]. However, protein mass spectrometry has multiple limitations such as high capital costs, limited sensitivity, and incomplete coverage of the proteome [5]. Consequently, traditional antibody-based techniques will likely continue to see extensive use in systems biology-based investigations. Such techniques include immunocytochemistry, immunoblotting, immuno-assays, immuno-electron microscopy, immuno-precipitation, and surface labeling for fluorescence-activated cell sorting.

In the ‘Best of All Possible Worlds’, antibodies to the protein products of all genes would be developed *a priori* and the investigator could order the ones required for a given study. Ultimately the economic marketplace may be able to achieve this. However, the present antibody marketplace offers antibodies directed to only a minority of proteins expressed in a given cell type, chiefly those that have been well studied. Furthermore, a broadly supported observation among biologists is that many commercial antibodies do not work well for their particular applications. Beyond this, there are several efforts hosted in academic environments aim to develop large sets of well-characterized antibodies directed to human proteins [6–8] and [http://commonfund.nih.gov/proteincapture/highlights.aspx]. Despite these efforts, it is likely that individual investigators will continue to need to develop new antibodies directed to specific tasks. This is particularly true when the experimental systems being utilized are derived from non-human species. Thus, a user-friendly set of procedures and tools for new antibody development is desirable. Here we describe an approach to the selection of peptide antigens for producing antibodies to specific target proteins, focusing on the identification of linear epitopes in disorganized regions of these proteins. There is a large literature on the use of three dimensional structure models to identify targets in organized domains of proteins, which are not discussed in this paper [9].

The first step in the production of a new antibody is the choice of immunogen. Immunogens for most new antibodies today are synthetic peptides, typically from 12–30 amino acids in length. Others are made from fusion proteins obtained by expressing them in bacteria or insect cells. This article focuses on the choice of amino acid sequences for generation of immunogens that are designed to optimize the properties of the resulting antibodies. The method for selecting immunizing peptides is independent on whether monoclonal or polyclonal antibodies are being made. In general, immunogenicity is not the only factor that needs to be taken into consideration. In order to have maximal utility, an antibody must be as specific as possible, ideally recognizing a single protein. Certain amino acid sequences are shared by multiple proteins so that use of these sequences to make the immunogen could result in an antibody that is not specific to the desired target. Furthermore, sometimes there is a need to use antibodies to recognize the same protein in multiple animal species, requiring that the immunizing sequence chosen is conserved among these species. In addition, there is a need to avoid regions that undergo PTMs like phosphorylation because such modifications can ablate an epitope, making the modified protein “invisible” to the antibody. Thus, the choice of the optimal immunogen involves evaluation of trade-offs among all of these factors (immunogenicity, specificity, conservation and presence/absence of PTMs) for candidate peptides. This comparison can be difficult unless aided by visualization tools that show all of these properties aligned with the primary sequence of the protein. To address this need we developed a software tool, *AbDesigner*, for our own antibody design projects and eventually produced an online version (http://helixweb.nih.gov/AbDesigner/) that has been widely used in the community [10]. The purpose of this article is to describe the features of *AbDesigner*, to compare it to other software tools that are currently available and to illustrate its use in the design of a particular antibody.

### Features of *AbDesigner*

*AbDesigner* accepts official gene symbol, Swiss-Prot accession number, or Swiss-Prot entry name of a protein from any of the following seven species: *Homo sapiens*, *Rattus norvegicus*, *Mus musculus*, *Drosophila melanogaster*, *Caenorhabditis elegans*, *Saccharomyces cerevisiae*, and *Arabidopsis thaliana* as an input. It then extracts the amino acid sequence and other supporting information of a given input protein from the Swiss-Prot protein database locally stored on the server for the subsequent analysis. Proteins from other species can also be analyzed by entering the FASTA amino acid sequence (with some limitations in analysis due to a lack of the server-side protein database for those proteins). The user can then specify a peptide length intended to be used for immunization and an epitope length used for the determination of uniqueness and conservation of a peptide as described below. The analysis by *AbDesigner* can be executed after the above parameters are filled.

**Figure 1:**
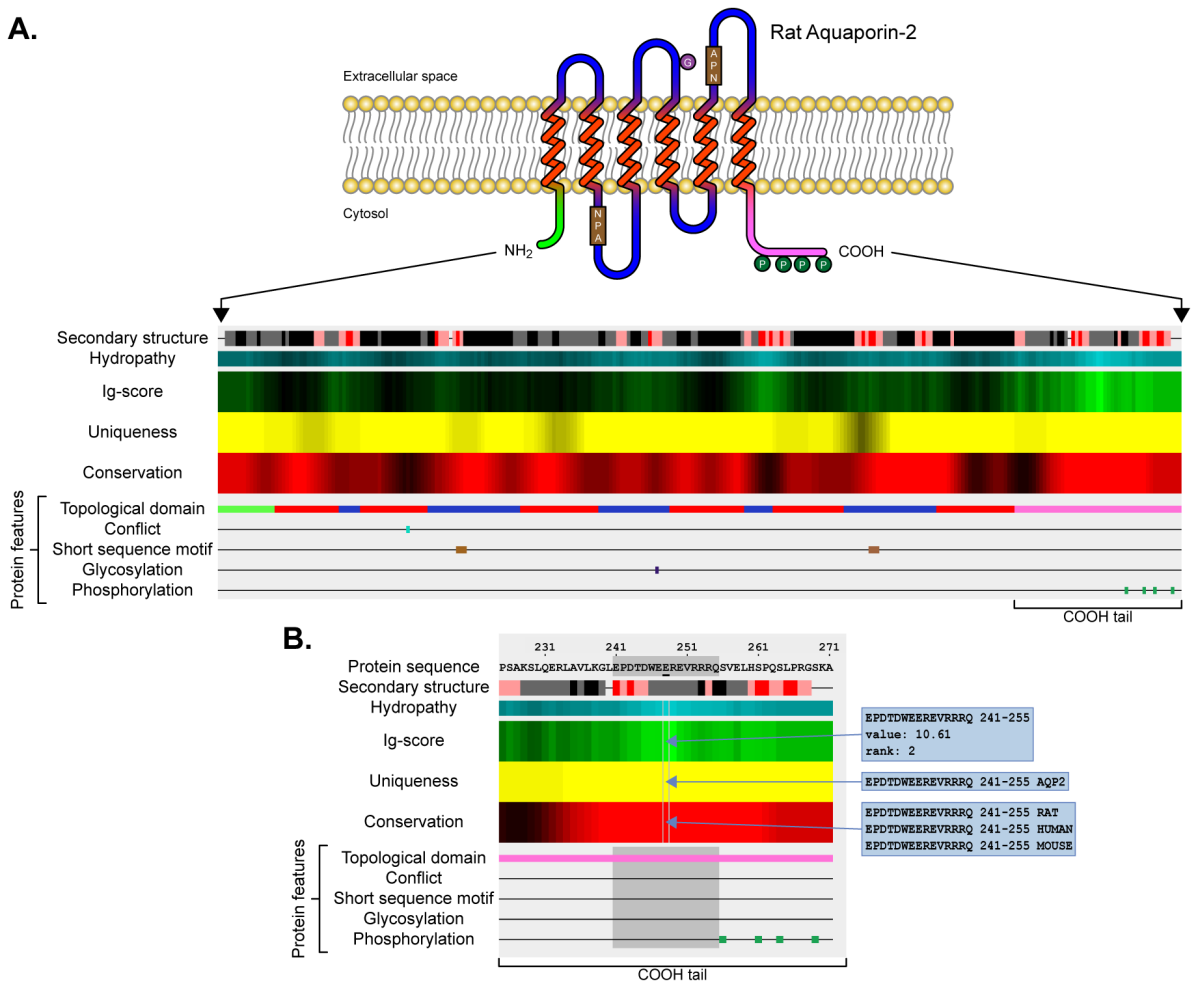
*AbDesigner* output **A)** *AbDesigner* analyzes the amino acid sequence of a protein of interest (apical membrane water channel, aquaporin-2 of rat with six membrane-spanning regions depicted in this example) and illustrates the immunogenicity score (Ig-score, in a green heat map), which is aligned with the calculated uniqueness score (in a yellow heat map), the calculated conservation score (in a red heat map), and protein features extracted from the corresponding Swiss-Prot record. By default, the highest Ig-score, uniqueness score, and conservation score are displayed in the brightest green, yellow, and red, respectively. The Kyte-Doolittle hydropathy index is shown in a cyan heat map (most hydrophilic displayed in the brightest cyan) and the Chou-Fasman secondary structure prediction is displayed by various colors (top row: alpha helix = gray, beta sheet = black, strong beta turn = red, or weak beta turn = pink). **B)** A magnified view of the carboxyl-terminal region of the graphical output of aquaporin-2 in A. When a peptide is selected by a mouse click, its sequence, scores, and features will be highlighted in gray. Further details for each element of the output can be viewed by mouse-over including the Ig-score value and rank as well as the multiple sequence alignments for representing the uniqueness and conservation.

Figure 1 elucidates the graphical output of *AbDesigner* on the analysis of an example protein, rat aquaporin-2 (Aqp2). Aqp2 is an apical membrane water channel with six membrane-spanning regions as depicted in Figure 1A (the topological cartoon is for demonstration purposes only, not shown in the real output). *AbDesigner* output illustrates several features of a protein relevant to the choice of a synthetic peptide sequence to be used as an immunogen in antibody production including hydropathy, secondary structure, immunogenicity (Ig-score), uniqueness, conservation among species, and other protein features e.g. topological domain, the presence or absence of PTMs, and etc. (Figure 1A). Ig-score is a product of the Kyte-Doolittle hydropathy index, the average Chou-Fasman conformational parameters of beta turn, and the tail bonus, see the appendix in Pisitkun et al. [10]. This output interactively responses to a mouse action and displays further details of each output element in a manner that allows the user to judge trade-offs for candidate peptide sequences with respect to multiple factors (Figure 1B). In this example, a peptide “EPDTDWEEREVRRRQ” in the carboxyl-terminal region of aquaporin-2 is selected. It has a high Ig-score (a value of 7 or more is considered high immunogenicity) and ranks second among all the peptides with equal length. The peptide is unique (epitope matching, across rat proteome, to only Aqp2) and well conserved among rat, human, and mouse. It also does not overlap with any known sites of PTMs (e.g. phosphorylation or glycosylation sites) or regions with conflicting sequence due to alternative splicing. Thus, this information reveals the proper characteristics of the peptide to the user and facilitates the selection process for a suitable immunogen.

### Comparison of *AbDesigner* to other software tools

Various computational methods have been developed to analyze different factors necessary for the determination of the choice of amino acid sequences for immunogen generation. These methods generally predict parameters that determine immunogenicity of linear epitopes of a protein such as hydropathy (e.g. by Kyte and Doolittle [11] and Hopp and Woods [12]), secondary structure (e.g. by Chou and Fasman [13] and Garnier et al. [14]), surface probability (e.g. by Emini et al. [15] and Janin [16]) and integrated antigenic index (e.g. by Jameson and Wolf [17] and Kolaskar and Tongaonkar [18]). The judgment of immunizing peptides also relies on the uniqueness of amino acid sequence which helps foresee the specificity of an antibody. On the other hand, the conservation of amino acid sequences among all species of interest aids the production of an antibody that recognizes a given protein in more than one species. The sequence alignment methods such as the basic local alignment search tool (BLAST) and multiple sequence alignment (MSA) algorithms are commonly used to assess the uniqueness and conservation of an immunogen. The information about other protein features e.g. PTMs also play an important part in the choice of the optimal immunogen. When multiple factors must be examined, it can be challenging to consider all of the relevant information from various sources using multiple software tools. An integration of these methods into one suite, as implemented in *AbDesigner*, assists the task. Table 1 compares *AbDesigner* to other existing integrated antibody design tools regarding accessibility, interactivity, comprised methods (based primarily on linear epitope prediction), and other features. *AbDesigner* is freely available as a web application that interactively exhibits information relevant to the choice of the optimal immunizing peptide, including integrated immunogenicity (antigenic) score, uniqueness (predictor of specificity), conservation (predictor of multispecies cross-reactivity), and relevant protein features such as PTMs, domain architecture, sites of sequence variation, and other regions or sites of interest extracted from the corresponding Swiss-Prot record. Another software tool that has many of the features reported in *AbDesigner* is the Epitope Choice Resource (EpiC) from the European Molecular Biology Laboratory [19]. In EpiC, however, various graphical data in an output of the software are not properly aligned and a corresponding peptide sequence for each predictive score is not displayed thus making it difficult for users to obtain the optimal sequence to be used for peptide production. Moreover, the assessment of uniqueness and conservation in EpiC (via BLAST) is not automatically performed and presented in real time.

## Example of Use of *AbDesigner*

In this review, we demonstrate the use of *AbDesigner* for the production of three successful antibodies against kidney disease-related proteins in human, viz. nephrin, podocin, and apolipoprotein L1. The results presented here are previously unpublished.

### Human nephrin antibody

**Figure 2:**
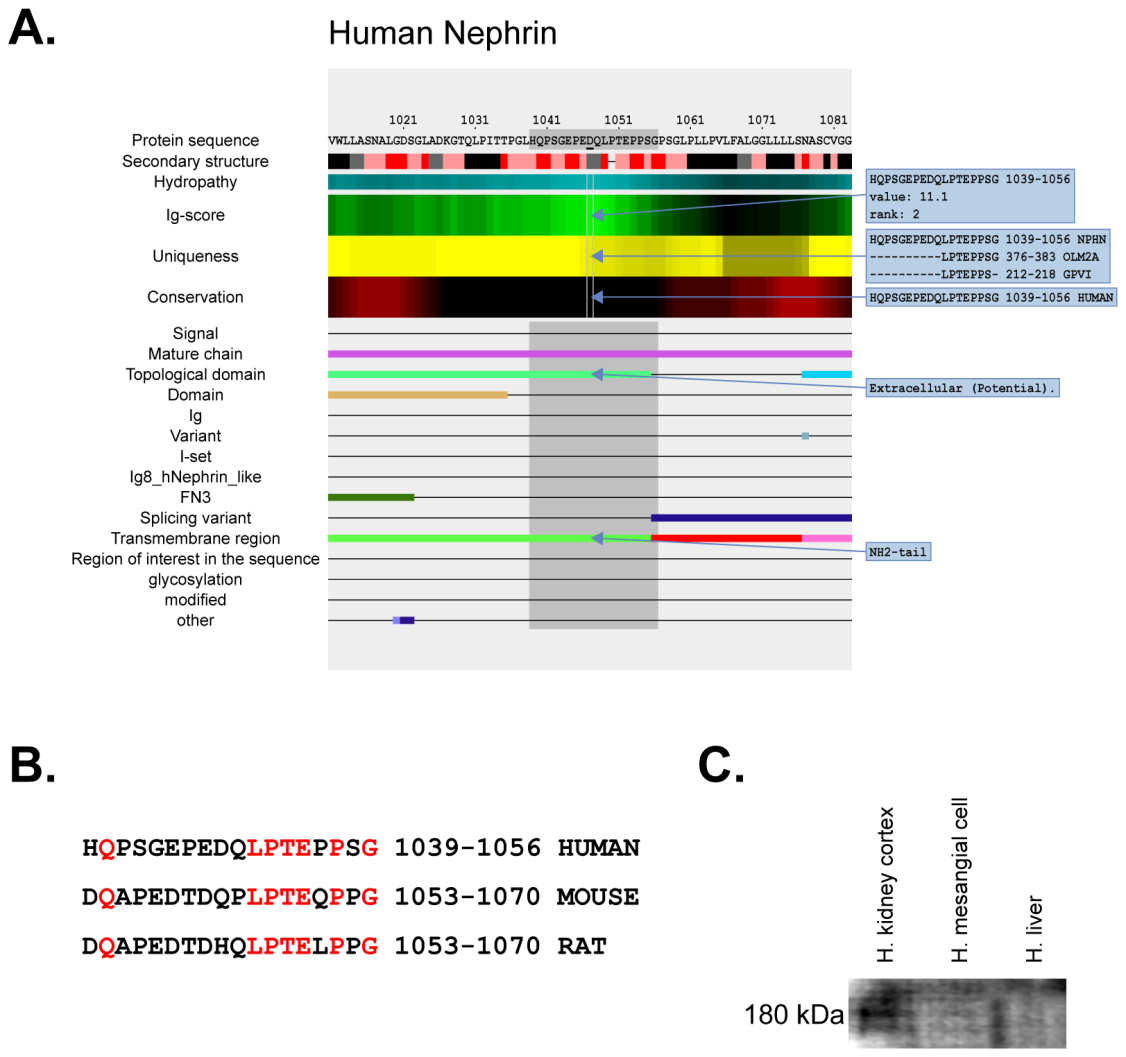
Human nephrin antibody **A)** A peptide corresponding to N-terminal amino acids 1039–1056 of human nephrin (highlighted in gray) was employed to make a new antibody. Input parameters for *AbDesigner*: Input Type = Gene Symbol-Human, Input = NPHS1, Peptide Length = 18, Epitope Length = 7. **B)** Alignment of the human peptide to mouse and rat nephrin. **C)** An immunoblot of human kidney cortex, mesangial cell, and liver samples probed with the K3 rabbit polyclonal antibody.

Nephrin is a single-pass type I membrane protein that is an important component of the blood plasma filtration mechanism in the glomerulus of the kidney. It is specifically expressed in podocytes of kidney glomeruli and its full sequence contains 1241 amino acids. Homozygous or compound heterozygous mutation in the gene encoding nephrin on chromosome 19q13.1 causes nephrotic syndrome type 1 (NPHS1), also known as Finnish congenital nephrosis. We intended to design the best antibody for human nephrin and accepted the fact that it might not cross-react with nephrin in other species. Figure 2A demonstrates the *AbDesigner* output (between amino acids 1011 to 1083) for human nephrin. An 18-mer peptide “HQPSGEPEDQLPTEPPSG” located in the extracellular N-terminal region next to the transmembrane region (shown as red bar) of the protein was selected for immunization. The peptide has a very high Ig-score and rank, is partially aligned with two other human proteins, and does not contain any known PTMs or mutation sites (thus the expected antibody can be used in the NPHS1 patients as well). However, this choice comes with the trade-off that the peptide sequence is not well conserved in mouse or rat nephrin as shown in Figure 2B. An extra cysteine “C” was added at the carboxyl-terminal end of the peptide for conjugation to carrier and construction of affinity-purification column. The peptide was conjugated to the carrier protein keyhole limpet hemocyanin (KLH) prior to immunization. The K3 rabbit polyclonal antibody was produced and successfully utilized to recognize the nephrin band at 180 kDa by immunoblotting of a human kidney cortex sample (Figure 2C). The specificity of the antibody was supported by a lack of signal in human mesangial cell and liver samples. The antibody does not recognize mouse nephrin as predicted (data not shown).

### Human podocin antibody

**Figure 3:**
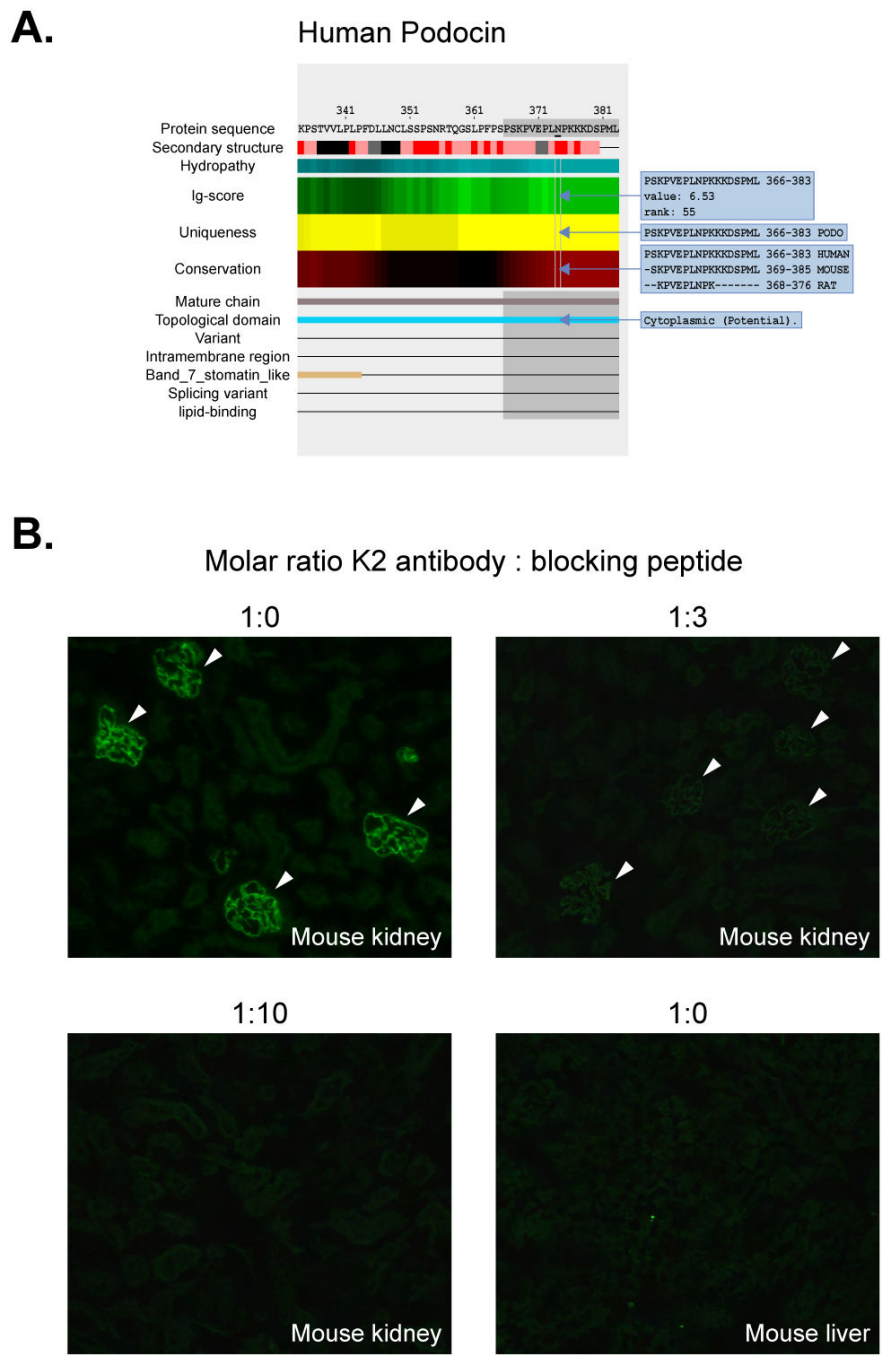
Human podocin antibody **A)** A carboxyl-terminal peptide (amino acids 366–383) of human podocin (highlighted in gray) was utilized for the production of a new antibody. Input parameters for *AbDesigner*: Input Type = Gene Symbol-Human, Input = NPHS2, Peptide Length = 18, Epitope Length = 7. **B)** Immunofluorescence staining of mouse frozen kidney and liver sections with the K2 rabbit polyclonal antibody, different molar ratios of the antibody and blocking peptide were used. Arrowheads indicate glomeruli with the distinctive ribbon-like pattern corresponding to podocyte staining.

Podocin is a peripheral membrane protein that is almost exclusively expressed in podocytes and interacts with nephrin. Its full sequence contains 383 amino acids. Homozygous or compound heterozygous mutation in the gene encoding podocin (*NPHS2*) on chromosome 1q25–q31 causes nephrotic syndrome type 2 (NPHS2). We proposed to design an antibody that can specifically recognize podocin across different species. Figure 3A displays the *AbDesigner* output (between amino acids 334 to 383) for human podocin. An 18-mer peptide “PSKPVEPLNPKKKDSPML” situated at the cytoplasmic carboxyl-terminus of the protein was chosen for immunization. The selected peptide has a moderate Ig-score, but is unique to this protein (epitope matching to only podocin), as well as being well conserved in mouse podocin, and partially conserved in rat podocin. It does not include any known PTMs or mutation sites. The peptide was synthesized with a cysteine added at the N-terminal end and conjugated to KLH prior to immunization. The K2 rabbit polyclonal antibody was generated. By immunofluorescence staining, in the absence of competitive blocking with the immunizing peptide, the antibody distinctively detected podocytes in a mouse frozen kidney section based on the characteristic ribbon-like pattern of the glomeruli (arrowheads in the left upper panel of Figure 3B). The antibody specificity was validated by a reduction in the fluorescence signal in mouse kidney when increasing molar ratios of the blocking peptide were used (Figure 3B). Staining of a mouse frozen liver section was negative as anticipated, further indicating specificity of the antibody. Notice that not only is the antibody specific but it also cross-reacts with the other species as expected. This example also shows that the suitable peptide does not need to have the best immunogenicity and emphasizes the use of multiple factors in the selection process.

### Human apolipoprotein L1 antibody

**Figure 4:**
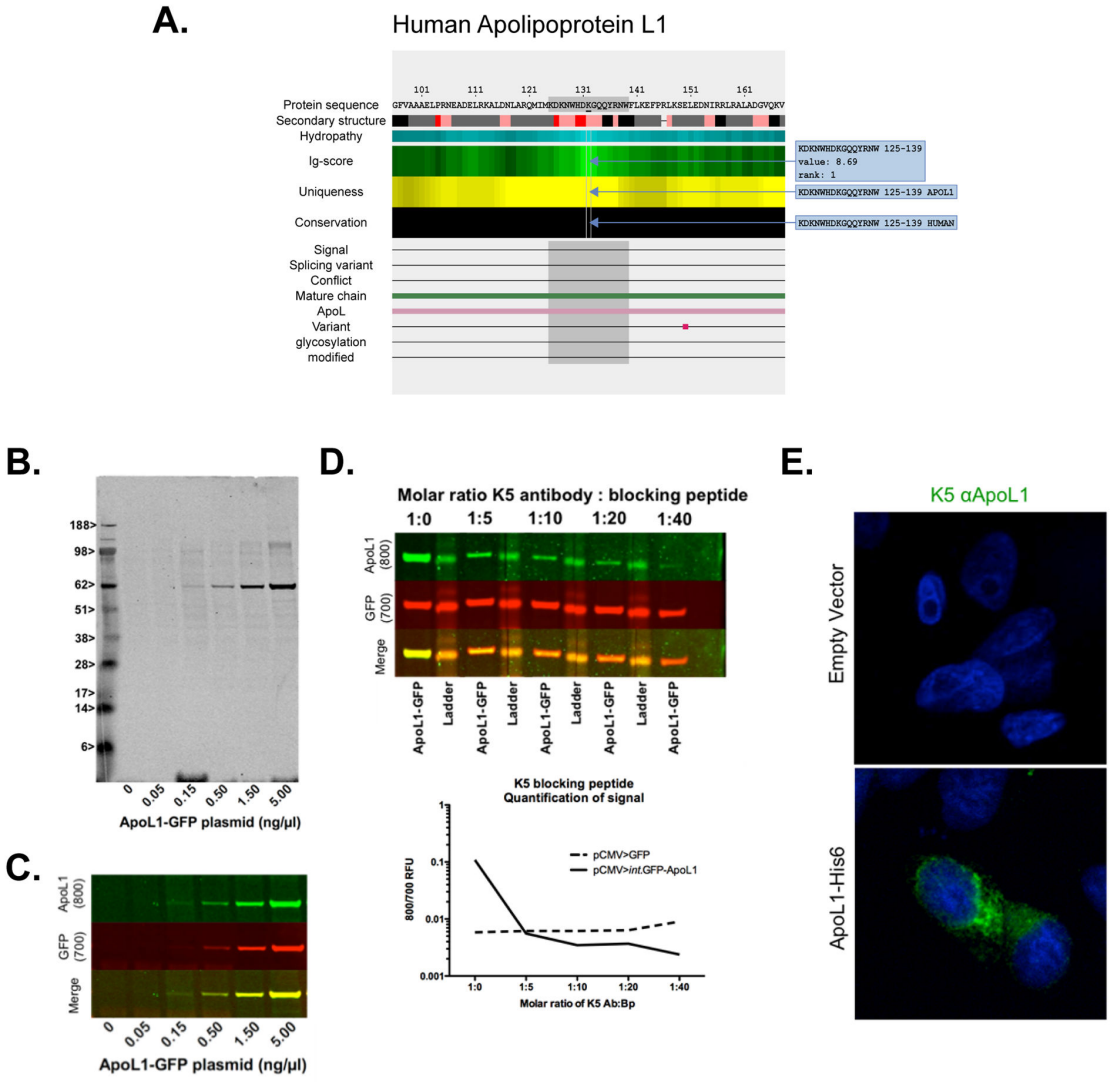
Human apolipoprotein L1 antibody **A)** A peptide corresponding to amino acids 125–139 of human apolipoprotein L1 or ApoL1 (highlighted in gray) was selected for generating a new antibody. Input parameters for *AbDesigner*: Input Type = Gene Symbol-Human, Input = APOL1, Peptide Length = 15, Epitope Length = 7. **B)** Immunoblotting of lysates from CHO cells transfected with pCMV>GFP and/or increasing amounts of pCMV>ApoL1-GFP plasmid DNA as shown was probed with the K5 rabbit anti-ApoL1 antibody. The 62.5 kDa band that intensifies with increasing plasmid amount is of the expected molecular weight for ApoL1-GFP protein. **C)** The membrane shown in (B) was blotted with mouse anti-GFP (700 nm channel) and the K5 rabbit anti-ApoL1 antibody (800 nm channel). The yellow band in the merged image indicates colocalization between the 700 nm and 800 nm channels at 62.5 kDa. **D)** Competitive blocking of the K5 antigen peptide was used in increasing ratio to the K5 antibody. **E)** Representative image from indirect immunofluorescence microscopy of HeLa cells transfected with 5 ng/μl pCMV>ApoL1-His6 or empty vector control and stained for ApoL1 using the K5 anti-ApoL1 antibody. No signal was observed in cells transfected with empty vector, whereas the pCMV>ApoL1 transfected cells displayed a robust cytoplasmic signal, particularly in a perinuclear location. The experiment was repeated four times with similar results.

Apolipoprotein L1 (ApoL1) is a secreted protein found in plasma. Its full sequence contains 398 amino acids. Variation in the *APOL1* gene on chromosome 22q12.3 confers susceptibility to focal segmental glomerulosclerosis, HIV-associated nephropathy, and hypertension-attributed kidney disease. This gene is unique to certain primates and thus conservation across species is not a concern in our design. Figure 4A illustrates the *AbDesigner* output (between amino acids 96 to 168) for human ApoL1. We selected a 15-mer peptide “KDKNWHDKGQQYRNW” with the highest Ig-score rank and specificity to the protein. The peptide does not carry any known PTMs or variant sites. The K5 rabbit polyclonal antibody was made using the KLH-conjugated peptide (with a cysteine added at the carboxyl-terminal end). Using ApoL1 transfected cells, the antibody was successfully validated by immunoblotting (Figure 4B and 4C), competitive blocking with the immunizing peptide (Figure 4D), and immunofluorescence microscopy (Figure 4E). This example demonstrates an undemanding immunogen design task facilitated by *AbDesigner*.

## Conclusion

*AbDesigner* is a freely available and fully integrated online application that analyzes and displays the attributes of a protein pertinent to the choice of a synthetic peptide sequence to be used as an immunogen in antibody production. In particular, the software allows the user to recognize trade-offs for candidate peptide sequences pertaining to multiple factors including immunogenicity, uniqueness, conservation, and the presence or absence of PTMs and other protein features. Use of *AbDesigner* enables the design of immunogenic antigens to generate antibodies that will (ideally) have specificity for a single protein (or if desired a protein family) and will recognize protein from one animal species (or if desired will recognize more than one species). *AbDesigner* has been well-received by researchers in academic and industrial institutes around the world with > 7,500 page visits (71% returning visitors) from the 5 major continents since February 2012 (reported by Google Analytics). In conclusion, we believe that *AbDesigner* will assist in the rapid and accurate design of peptide-directed antibodies to analyze protein abundance, localization, PTMs, protein-protein interactions, and function. As it may be feasible to generate mono-specific antibodies against entire suites of proteins, such as all members of particular protein families or of particular functional classes, this approach will facilitate comprehensive studies of proteins when carried out at a systems level.

## Figures and Tables

**Table 1:** Comparison of NHLBI-*AbDesigner* to other integrated antibody design tools.

Features/Tools	NHLBI *AbDesigner*	EpiC	IEDB	DNASTAR Protean	Gene Inspector	Antigen Profiler
**Freely available**	+	+	+	−	−	−
**Web application**	+	+	+	−	−	−
**Interactive graphical user interface**	+	+	+	+	Not tested	−
**Hydropathy**	+	+	+	+	+	+
**Secondary structure**	+	−	+	+	+	−
**Surface probability**	−	+	+	+	+	−
**Integrated antigenic score**	+	+	+	+	−	+
**Specificity assessment**	+	+	−	−	−	+
**Multi-species recognition assessment**	+	+	−	−	−	+
**Other protein features e.g. PTMs**	+	+	−	+	+	+

URLs: NHLBI-*AbDesigner* = http://helixweb.nih.gov/AbDesigner/, Epitope Choice Resource (EpiC) = http://bioware.ucd.ie/epic/, Immune Epitope Database and Analysis Resource (IEDB) = http://tools.immuneepitope.org/tools/bcell/iedb_input, DNASTAR Protean = http://www.dnastar.com/t-protean.aspx, Gene Inspector = http://www.textco.com/gene-inspector.php, Antigen Profiler = http://www.pierce-antibodies.com/custom-antibodies/peptide-design-antigen-profiler.cfm
